# Fabrication of Eco-Friendly Superabsorbent Composites Based on Waste Semicoke

**DOI:** 10.3390/polym12102347

**Published:** 2020-10-14

**Authors:** Yongsheng Wang, Yongfeng Zhu, Yan Liu, Aiqin Wang

**Affiliations:** 1 Key Laboratory of Clay Mineral Applied Research of Gansu Province, Center of Eco-material and Green Chemistry, Lanzhou Institute of Chemical Physics, Chinese Academy of Sciences, Lanzhou 730000, China; wysh0304@126.com (Y.W.); zhuyf851013@163.com (Y.Z.); liuyanlwb@foxmail.com (Y.L.); 2 Center of Materials Science and Optoelectronics Engineering, University of Chinese Academy of Sciences, Beijing 100049, China

**Keywords:** superabsorbent composites, semicoke, water absorbency

## Abstract

A series of novel superabsorbent composites of poly(acrylic acid)/semicoke were prepared by polymerization of acrylic acid using ammonium persulphate as initiator, *N*,*N*′-methylenebisacrylamide as crosslinker and semicoke which was the by-product of coal carbonizing as the inorganic components. FTIR and SEM analysis indicated that the superabsorbent composites had been successfully polymerized and the semicoke participated in construction of the 3D polymeric network. Meanwhile, the effects of initiator, crosslinker, semicoke, and neutralization degree, as well as the pH value, were investigated, and the results showed that superabsorbent composites containing 10% of semicoke possessed the maximum water absorbency of 584 g/g in distilled water and 75 g/g in 0.9% NaCl solution. The superabsorbent composites kept the high water absorbency within a wide pH range of 4–11, and still exhibited better re-swelling capability even after seven times. The superabsorbent composite with its excellent performance is a potential water-retaining agent used in agriculture.

## 1. Introduction

Superabsorbent hydrogels are three-dimensionally crosslinked networks that can absorb and retain possibly large amounts of water in the aqueous solvent [1]. Since the first superabsorbent hydrogel was available in the 1970s by the American department of agriculture [2], superabsorbent hydrogels have been promising applications in various fields such as agriculture and horticulture [3,4], adsorbents [5,6], materials for personal hygiene products [7,8], drug delivery systems [9,10], etc. for their excellent water-absorption and retention capabilities. Especially in agriculture, the superabsorbent hydrogels have been widely sought after in society because of their water storage function and slow release effect.

However, the superabsorbent hydrogels prepared by simply using fossil products such as acrylic acid (AA), have the downsides of high cost, consequently restricting its applications in agriculture. A large number of researches have indicated that the introduction of a certain amount of inorganic substances into the polymeric structure of superabsorbent hydrogels cannot only significantly increase its water absorption rate, but also effectively reduce the product cost. Therefore, the superabsorbent composites, which incorporate the inorganic materials including silicate mineral and metallic ions such as attapulgite [11,12], kaolinite [13,14], bentonite [15,16], titanium dioxide [17,18], zinc oxide, and aluminum ions [19] into the superabsorbent composites have attracted researchers’ widespread attention.

In recent years, with the concept of a “recycling economy” embedded deeply in human nature, the using of wastes as inorganic components for preparation of superabsorbent composites has become a new research tendency, with the purpose of realizing the substance circulation. These wastes applied in the superabsorbent composites include industrial wastes (waste polystyrene [20,21,22], polyacrylonitrile fiber wastes [23,24], etc.) and crop wastes (flax yarn wastes [25,26], corn stalks [27], etc.). For instance, Ismail et al. synthesized superabsorbent composites through emulsion polymerization of waste polystyrene starch, as well as acrylic acid, and the maximum water absorbency reached 500 g/g in distilled water [20]. Zhang et al. developed the eco-friendly flax yarn waste/polyacrylic acid superabsorbent composites, the water absorbency reaching a maximum of 490 g/g in rainwater and 90 g/g in 0.9 wt% NaCl solution [25].

Oil shale semicoke (SC) is a byproduct from the thermal processing of oil shale consisting of the stable carbonic matter and other inorganic minerals, including quartz, kaolinite, hematite, carbonate, and others [28]. Due to the current technology not fully utilizing the SC by high value-added approach, many problems afflict the coal and petrochemical enterprises seriously, such as the high waste of land resources and potential threat to the environment [29,30]. Encouragingly, a great deal of research has investigated the utilization of SC in several fields in recent decades, and provided many basic datum and methods for effective recycling of SC. For instance, Nicolini et al. recommended the addition of SC in soils to degrade the polycyclic aromatic hydrocarbons (PAHs), thus allowing the future using of SC as an agricultural soil conditioner [31]. The application of SC as alternative fuel in iron ore sintering was studied by Luo et al., and showed that coke breeze could be substituted with SC without affecting the sintering [32]. Wang et al. loaded the V_2_O_5_ onto the activated SC (ASC) via impregnation method and used for low temperature selective catalytic reduction of NO_X_ with NH_3_. The prepared V_2_O_5_/ASC catalyst helped to improve conversion rate and N_2_ selectivity [33]. For all this, the overall utilization amount of SC is still limited, so the novel transformation path of SC waste to new materials still needs to be explored.

It is worth noting that the content of inorganic mineral and carbonic matter of some SC components is about 70% and 30%, respectively [34]. The carbonic matter of SC is mainly composed of PAH which contain a large number of active functional groups, such as hydroxyl group or carboxyl group. These functional groups could replace inorganic minerals to form a favorable interaction with the hydrogel’s backbone since silicate minera particles did not have proper surface functionalities. Furthermore, the introduction of inorganic minerals not only significantly improved its water absorption and stability, but also effectively reduced the product cost. Thereby, the application of SC into the superabsorbent composites, like other inorganic minerals, is entirely feasible and has broad prospects. So in this study, we designed and prepared a series of superabsorbent composites by polymerization of AA as the presence of SC, using ammonium persulphate (APS) as initiator and *N*,*N*’-methylene-bisacrylamide (MBA) as crosslinker in aqueous solution. The affecting factors of the water absorbency, such as content of APS, MBA, and SC, as well as neutralization degree were all investigated. The swelling kinetics, water-retention capacity, and the reswelling capability were also tested carefully. We expected the experiment result can be an effective reference for application of the SC in superabsorbent composites and agriculture ultimately.

## 2. Materials and Methods

### 2.1. Materials

Oil shale semicoke micropowder (Yaojie Coal and Electricity Group Co., Ltd., Gansu, China, XRF composition analysis: SiO_2_ 54.85%, Fe_2_O_3_ 14.12%, Al_2_O_3_ 21.70%, MgO 1.55%, CaO 2.48%, K_2_O 1.45%), milled through 300-mesh screen before using; Acrylic acid (AA, chemically pure) was purchased from Shanghai Wulian Chemical Factory, (Shanghai, China); Ammonium persulfate (APS, analytical grade) was obtained from Xi’an Chemical Reagent Factory, (Xi’an, China) and used after the recrystallization. *N*,*N*′-Methylene bisacrylamide (MBA, chemically pure) was supplied from Shanghai Chemical Reagent Factory (Shanghai, China); other chemical reagents were all analytical grade and utilized as obtained without further purification. All the solutions were prepared with distilled water.

### 2.2. Preparation of the Superabsorbent Composites

Typically, 800 mg of SC (10 wt% to the total amount of AA and SC) was dispersed in 30 mL of distilled water and then transferred to a 250 mL three-necked flask equipped with a stirrer, a reflux pipe and a nitrogen line while constantly stirring to disperse SC uniformly. Subsequently, the dispersion was heated to 75 °C and kept for 30 min under the nitrogenous atmosphere to remove oxygen. Later, 5 mL of aqueous solution containing 365 mg of APS (1.60 mol% of AA) was dropped into the three-necked flask and continuously stirred for 2 min. After that, a mixing solution containing 7.2g of AA (neutralized 60% with 8.0 mol/L NaOH) and 61.7 mg of MBA (0.4 mol% of AA) was dropped into the reaction system. The oil bath was held constantly at 75 °C for 2 h to complete the reaction and the entire process of reaction was implemented under the atmosphere of nitrogen. The resultant products were dried at 90 °C to a constant weight. A series of superabsorbent composites with different content of SC, crosslinker, initiator, and acrylic acid were prepared via the above procedure. All samples were milled and passed through a 40–60 mesh sieve. The yields of simples prepared in various conditions are all listed in Table S1.

### 2.3. Water Absorbency and Swelling Kinetics Measurements

About 50 mg of the dried superabsorbent composites were immersed in 400 mL of distilled water or 0.9 wt% NaCl solutions with various pH values ranging from 2 to 14 for 4 h to reach the swelling equilibrium at room temperature. The superabsorbent composites were filtered with 100 mesh, and the swollen samples were drained under gravity for 10 min until there was no redundant water. The water absorbency *Q*_eq_ (g/g) was calculated by Equation (1) as follows:

*Q*_eq_ = (*W*_2_ − *W*_1_)/*W*_1_, (1)

where *W*_1_ (g) and *W*_2_ (g) are the weights of the dry sample and swollen sample, respectively. The swelling kinetics were investigated by measuring the water absorbency of 50 mg superabsorbent composites in 400 mL of distilled water or 0.9 wt% NaCl solution in different immersion times (10 min, 15 min, 30 min, 45 min, and 60 min), and the water absorbencies (*Q*_eq_) (g/g) were calculated according to Equation (1).

### 2.4. Water Retention and Reswelling Capability Measurements

The water retention capability was tested via the following methods. Pre-weight dry superabsorbent composites (50 mg) were immersed in 400 mL of distilled water or 0.9 wt% NaCl solution to reach the swelling equilibrium at room temperature. The superabsorbent composites were filtered with 100 mesh, and the swollen samples were drained under gravity for 2 min until there was no redundant water. The water-absorbed superabsorbent composites were weighted and placed in petri dishes at room temperature. The water retention properties at room temperature were calculated v the following formula:

Water retention = *Q*_t_/*Q*_i_, (2)

where *Q*_t_ is the weight of superabsorbent composites at time “*t*” and was calculated by Equation (1), *Q*_i_ is the initial weight of swollen superabsorbent composites. The reswelling of superabsorbent composites was investigated by measuring the water absorbency of 50 mg in 400 mL of distilled water until reaching the swelling equilibrium. The water absorbency of superabsorbent composites was calculated by Equation (1). The swollen sample was dried in a 90 °C oven to constant weight. We repeated the above process seven times and calculated the water absorption capacity of each swelling state.

### 2.5. Characterization

The polymerization structure of superabsorbent composites were analyzed with the FTIR in the wave number region of 4000–400 cm^−1^ using a Fourier transform infrared spectrometer (Nicolet NEXUS FTIR spectrometer, Thermo Fisher Scientific, Wilmington, DE, USA) by KBr pellet method. The morphology of the superabsorbent composites was observed with the Field Emission Scanning Electron Microscope (FE-SEM, JSM-6701F, JEOL, Tokyo, Japan) after coating the samples with gold film. The X-ray diffraction (XRD) pattern of crystal phase was collected in the range of 2θ = 3–80°, using a SmartLab SE multifunctional X-ray diffractometer (Rigaku Co., Tokyo, Japan). The thermal behavior was performed using a STA449C thermogravimetric analyzer (NETZSCH Co., Ltd., Selb, Germany) at a heating rate of 10 °C/min, in a temperature range from 100 to 700 °C under the synthetic air (component, O_2_:N_2_ = 1:4; flow rate, 50 mL/min). The Energy Dispersive Spectrometer (EDS, JSM-5600LV, JEOL, Tokyo, Japan) was performed.

## 3. Results and Discussions

### 3.1. FTIR Analysis

**Figure 1:**
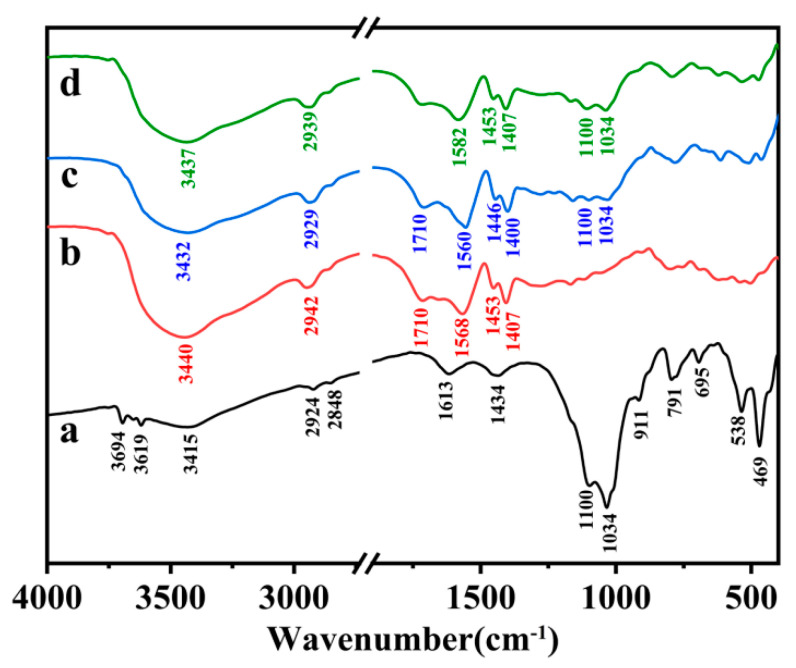
FTIR spectra of (**a**) SC, (**b**) PAA, (**c**) PAA/SC (10 wt%) and (**d**) the physical mixture of PAA with SC (*m*/*m* = 10).

The FTIR spectra of SC, PAA, PAA/SC (10 wt%) and the physical mixture of PAA with SC (*m*/*m* = 10) were shown in Figure 1. For the SC (Figure 1a), the absorption peaks at 1034 cm^−1^, 791 cm^−1^, 695 cm^−1^, 538 cm^−1^, 469 cm^−1^ were attributed to the Si–O, Si–O–Si and Si–O–Al, respectively. More importantly, the peaks at 1100 cm^−1^, 3694 cm^−1^ and 3619 cm^−1^ assigned to the stretching vibration of the apical Si–O group, as well as the stretching vibration of O-H in kaolinite [13,27], suggested the SC contained the silicate mineral of kaolinite. The XRD characterization also verified the kaolinite of in SC (Figure S1, Supplementary Material). In addition, The SC also contained the carbonic matter, and the content was 27.3% based on the TG characterization (Figure S2, Supplementary Material). In the FTIR, the characterization peaks appearing at 2924 cm^−1^ and 2848 cm^−1^ were symmetric and asymmetric stretching vibration towards –CH_2_, characterization peak at 1613 cm^−1^ and 1434 cm^−1^ were -OH or the asymmetric stretching of –COO^−^ and the scissoring vibration of –CH [35]. They were derived from the carbonic matter, which was mixed with the silicate mineral of kaolinite to form a SC [28]. After the polymerization reaction, the C=O stretching vibrations at 1710 cm^−1^ (Figure 1b), assigned to carboxylic group of un-neutralized PAA, appeared. Besides, the new peaks at 1568 cm^−1^ and 1407 cm^−1^ were related to the asymmetric and symmetric –COO^−^ stretching vibrations for carboxylate salt of PAA, indicating that the polymerization reaction was successful. Compared with infrared spectra of SC and PAA, the characterization peaks of PAA all appeared in the FTIR of PAA/SC, but the absorption bands at 1613 cm^−1^ (–OH or –COO^−^ asymmetric stretching of SC), 1568 cm^−1^ and 1407 cm^−1^ (–COO^−^ asymmetric and symmetric stretching of PAA) shifted to 1560 cm^−1^ and 1400 cm^−1^ (Figure 1c). Forthermore, the characterization peaks of PAA/SC significantly shifted compared to infrared spectra of the physical mixture of PAA with SC (Figure 1d), suggested graft polymerization between PAA and SC through the hydroxyl or carboxyl group in carbonic matter of SC, and the SC participated in the construction of the 3D polymeric network.

### 3.2. SEM Analysis

**Figure 2:**
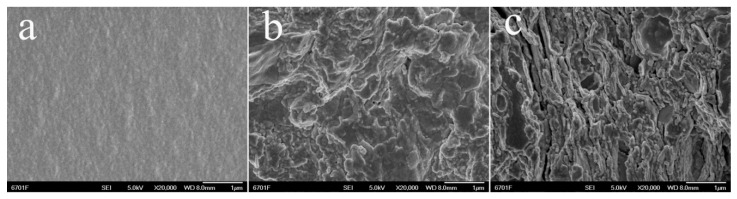
SEM micrographs of PAA/SC prepared by different content of SC: (**a**) 0 wt%, (**b**) 10 wt% and (**c**) 18 wt%.

The surface morphologies of superabsorbent composites towards PAA, PAA/SC (10 wt%) and PAA/SC (18 wt%) were shown in Figure 2. As can be seen, a comparatively smooth, dense and tight surface was observed to the PAA (Figure 2a). When the SC was introduced, the superabsorbent composites of PAA/SC (10 wt%) and PAA/SC (18 wt%) exhibited a relatively coarse and loose pleat surface (Figure 2b,c). The superabsorbent composite containing 10 wt% of SC showed lots of folds on the surface (Figure 2b). With the increase of SC content to 18 wt%, the surface roughness was obviously improved and the book-like structure was obviously found. Besides, some gaps also appeared in the surface of the composite containing 18 wt% of SC (Figure 2c). The corresponding elemental mapping was conducted and results revealed the homogeneous distribution of C, O, Si, Al and Fe over the entire structure of the obtained PAA/SC (10 wt%) (Figure S3, in Supplementary Material). This result proved the SC had finely dispersed and was embedded into the matrix of the PAA network. In addition, the SC had a role to relax the chain entanglement of PAA, which is similar to the clay of kaolinite, attapulgite, and so on [14,36]. The loose surface facilitates the permeation of water into the polymeric network and increases water absorption.

### 3.3. Water Absorbency

#### 3.3.1. Effect of Initiator Content on Water Absorbency

**Figure 3:**
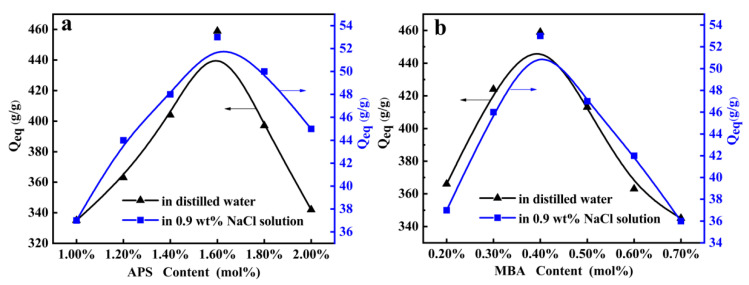
(**a**) Effects of APS content on the water absorbency (7.2 g AA, 800 mg SC, 0.4 mol% MBA and neutralization degree 70% of AA were selected and conditional screening of APS usage was conducted under nitrogen atmosphere); (**b**) Effects of MBA content on the water absorbency (7.2 g AA, 800 mg SC, 1.60 mol% APS and neutralization degree 70% of AA were selected and conditional screening of MBA usage was conducted under nitrogen atmosphere).

The effect of the initiator APS content on the water absorbency of the superabsorbent composites in distilled water and 0.9% NaCl solution as shown in Figure 3a (7.2 g AA, 10 wt% SC, 0.4 mol% MBA and neutralization degree 70% of AA were selected, and conditional screening of APS usage was conducted under nitrogen atmosphere). It can be seen that the water absorbency of the superabsorbent composites increased with the increase of the APS content, and reached an optimal value of 459 g/g in distilled water and 53 g/g in 0.9% NaCl solution as increasing the APS content to 1.6 mol%, respectively. Further increasing the content of APS, water absorbency of the superabsorbent composites began to decline. The polymerization reaction began from the decomposing of APS at the polymerization temperature. When the content of APS was lower than 1.6 mol%, a large number of free-radical sites on the polymer macromolecular chain may not form effectively, which limits the reaction process of chain transfer and the growth of grafting polymerization chain. Therefore, increasing the content of APS will produce more free-radical sites and extend the three-dimensional network, consequently improving the water absorbency of the superabsorbent composites. However, with further increasing of the APS content, the excess radicals cause the bimolecular collision termination step, thereby shortening the average length of the water absorption chain, and resulting in a decrease of water absorbency [37,38].

#### 3.3.2. Effect of Crosslinker Content on Water Absorbency

Figure 3b (7.2 g AA, 10 wt% SC, 1.60 mol% APS and neutralization degree 70% of AA were selected and conditional screening of MBA usage was conducted under nitrogen atmosphere) showed the effect of the MBA content on the water absorbency of superabsorbent composites. With the MBA content increasing from 0.4 mol% to 0.7 mol%, the water absorbency dropped rapidly from 460 g/g to 342 g/g in distilled water and from 53 g/g to 36 g/g in 0.9 wt% NaCl solution, respectively. The excess of crosslinker resulted in the generation of more crosslink points and an increase of the crosslink density, which decreases the gel network space left for holding water to enter, and causes the reduced water absorbency. The effect of the MBA for the water absorbency of the superabsorbent was quantitatively analyzed with Flory’s theory, as presented in Equation (3) [39]:

*Q*_eq_ = *kC*^−n^, (3)

where *Q*_eq_ is equilibrium water absorbency; *C* is the concentration of MBA; *k* and *n* are power law constants for an individual superabsorbent, which can be obtained from the curve fitted with Equation (3). As known from the calculation of PAA/SC superabsorbent composites, a power law relation between *Q*_eq_ and *C* was as follows: *Q*_eq_ = 14.96 *C*^−0.4678^ in distilled water and 3.98 *C*^−0.5363^ in 0.9 wt% NaCl solution, respectively. However, lower MBA content did not necessarily mean higher water absorption capacity. When the MBA content was below 0.4 mol%, the number of effective crosslinking points in the reaction system had decreased and the three-dimensional hydrophilic network of the superabsorbent composites couldn’t be formed efficiently. As a result, the soluble components in the superabsorbent composites increased, but the water absorption decreased [40].

#### 3.3.3. Effect of Neutralization Degree on Water Absorbency

**Figure 4:**
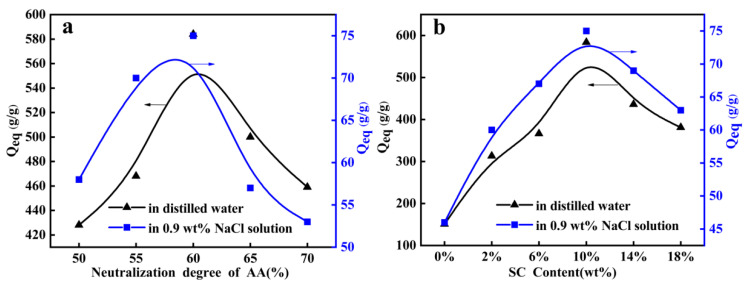
(**a**) Effects of the neutralization degree on the water absorbency (7.2 g AA, 800 mg SC, 0.4 mol% MBA and 1.6 mol% MBA were selected and conditional screening of neutralization degree of AA was conducted under nitrogen atmosphere); (**b**) Effects of SC content on the water absorbency (7.2 g AA, 0.4 mol% MBA, 1.60 mol% APS and neutralization degree 60% of AA were selected and conditional screening of SC usage was conducted under nitrogen atmosphere).

Figure 4a showed the effect of the neutralization degree on the water absorbency of superabsorbent composites. As can be seen in Figure 4a (7.2 g AA, 10 wt% SC, 0.4 mol% MBA and 1.6 mol% MBA were selected and conditional screening of neutralization degree of AA was conducted under nitrogen atmosphere), the water absorbency of the superabsorbent composites increased with the increase of neutralization degree of AA. As the neutralization degree of AA exceeded the critical value 60%, the water absorbency of the superabsorbent composites appeared to decrease. This trend can be attributed to generating the negatively charged carboxylate groups (–COO^−^) and introduction of sodium ions (Na^+^). The number of the negatively charged carboxylate groups in the gel network increased after neutralizing AA with NaOH, which resulted in the increase of the osmotic pressure difference between the gel network and the external solution. On the other hand, the negatively charged carboxylate groups attached to the polymer chains set up an electrostatic repulsion, which tended to expand the network of the swollen superabsorbent composites. In addition, the hydrogen bonds weakened interaction among the original –COOH groups with a decreasing of the proportion of –COOH group and leading to the decrease of effective crosslinking density. However, further increasing of the neutralization degree caused more sodium ions (Na^+^) to react with carboxylate groups (–COO^−^), and reduced the electrostatic repulsion, consequently presenting a decrease of water absorbency [41].

#### 3.3.4. Effect of Semicoke Content on Water Absorbency

The introduction of SC can change the composition and structure of superabsorbent composites, thus affecting their water absorbency. As shown in Figure 4b (7.2 g AA, 0.4 mol% MBA, 1.60 mol% APS and neutralization degree 60% of AA were selected and conditional screening of SC usage was conducted under nitrogen atmosphere), with the SC content increasing from 0 wt% to 10 wt%, the water absorbency increased rapidly from 150 g/g to 584 g/g in distilled water and from 46 g/g to 75 g/g in 0.9 wt% NaCl solution, respectively. When the SC content was over 10 wt%, the water absorbency of the superabsorbent composite gradually decreased. The reason may be the SC participate in construction of the three-dimensional network and relieved the entanglement of polymer chains and weakened the hydrogen bonding interaction between the functional groups [27,42]. In addition, the introduction of SC could prevent the polymer network structure from collapsing effectively during the drying process, so that the water absorbent of superabsorbent composites could be improved. However, with the increase of SC content, the activity of hydroxyl or carboxyl groups in the humus of SC was weakened, which affected the graft polymerization. And an excess of SC particles was filled in the polymer network structure by the physical form and resulted in the decrease of gel hydrophilicity. On the other hand, physical filling of SC will block the polymer networks structure and lead to decrease the water absorbency of the superabsorbent composites. It is worth noting that the water absorbency of the superabsorbent composites of the introduction SC significantly outperformed the blank sample.

#### 3.3.5. Effect of pH on Water Absorbency

**Figure 5:**
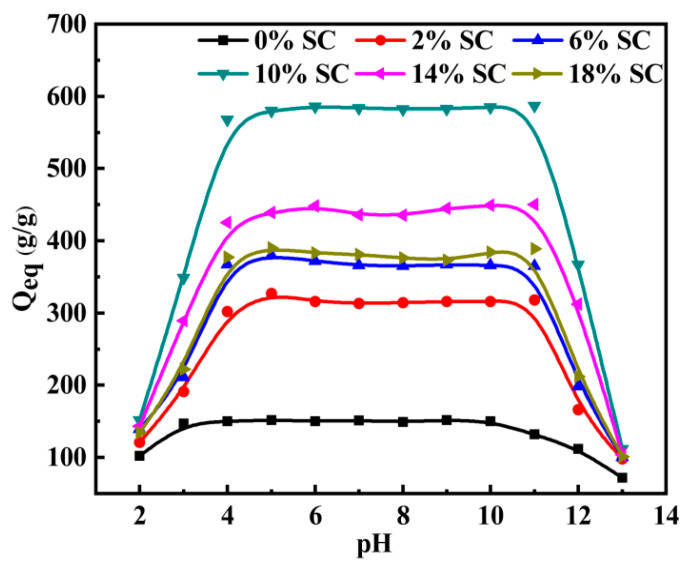
Effects of the pH on the water absorbency of PAA/SC with various SC content (7.2 g AA, 0.4 mol% MBA, 1.60 mol% APS and neutralization degree 60% of AA were selected and prepared superabsorbent composites with SC content 0 wt%, 2 wt%, 6 wt%, 10 wt%, 14 wt% and 18 wt%, respectively).

The swelling properties of the PAA/SC in the solutions with various pH values were evaluated and shown in Figure 5. It can be seen that the water absorbency of PAA/SC almost kept constantly in the pH range of 4–11, but rapidly increased with increasing pH in the range of 2–4 and decreasing pH from 11 to 13. At strong acidic solution (pH < 4), most of the carboxylate groups were protonated to form –COOH groups, the hydrogen bonds formed among –COOH groups induced polymer and polymer interactions that predominate over polymer and water interactions, which also reduced the water absorbency [43,44]. Meanwhile, limited anion–anion electrostatic repulsion may also lead to a decrease in absorbency. At basic pH (pH > 11), the increase of ionic strength of the external solution caused a rapid decrease of ion osmotic pressure and an increase abruptly of screening effects of Na^+^. In the pH range of 4–11, because of the buffer action of –COO^−^ and –COOH groups in aqueous solution, the water absorbency of PAA/SC kept almost constantly equal to their equilibrium water absorbency [45]. This feature of wide pH range towards PAA/SC will facilitate its application in various types of soil.

#### 3.3.6. Swelling Kinetics

**Figure 6:**
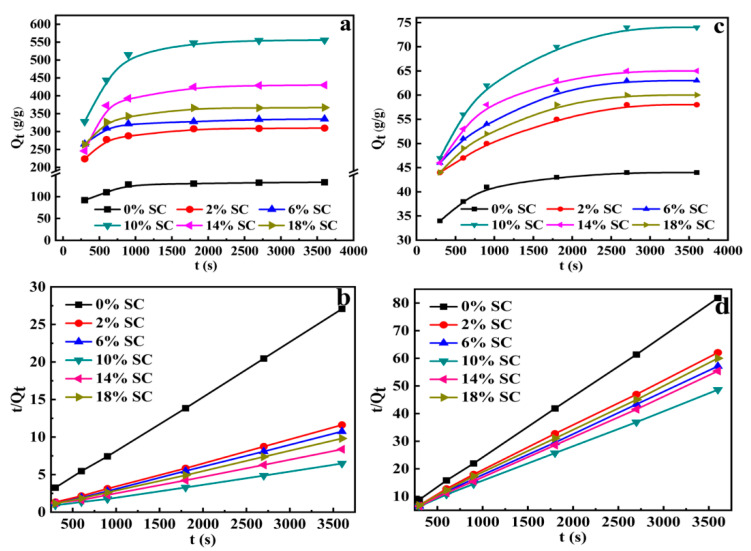
(**a**) Swelling kinetic curves in distilled water; (**b**) *t*/*Q*_t_ vs. t graphs in distilled water; (**c**) swelling kinetic curves in 0.9 wt% NaCl solution; (**d**) *t*/*Q*_t_ vs. t graphs in 0.9 wt% NaCl solution (7.2 g AA, 0.4 mol% MBA, 1.60 mol% APS and neutralization degree 60% of AA were selected and prepared superabsorbent composites with SC content 0 wt%, 2 wt%, 6 wt%, 10 wt%, 14 wt% and 18 wt%, respectively).

The introduction of SC into superabsorbent composites can affect the composition of the gel and swelling kinetics. As shown in Figure 6, the effect of the solution on the swelling behaviors of superabsorbent composites of different SC content were measured in distilled water and in 0.9 wt% NaCl ssolution. It can be seen that the swelling rate of the superabsorbent composites in distilled water and salt solution sharply increased within 1800 s and 2700 s, respectively. Then the swelling rate began to level off, and the swelling kinetic curves became flatter. The swelling kinetics behavior of the PAA/SC was evaluated by means of the Scott’s second-order Equation (4) [46]:

*t*/*Q*_t_ = 1/*K*_s_*Q*_∞_^2^ + *t*/*Q*_∞_, (4)

where *Q*_t_ is the water absorbency at a given time t; *K*_s_ is swelling rate constant; *Q*_∞_ is the equilibrium water absorbency and *K*_is_ = *K*_s_*Q*_∞_^2^ is the initial swelling rate of the superabsorbent composites. Based on the experimental data, the plots of *t*/*Q*_t_ vs. t were given perfect straight lines with good linear correlation coefficient, indicating that the swelling of the PAA/SC fit well with the Scott’s swelling theoretical model. Also, by fitting experimental data using Equation (4), the swelling kinetic parameters including *K*_s_, *Q*_∞_ and Kis can be calculated by the slope and ordinate intercept of lines [47]; results are listed in Table 1 and Table 2.

According to the obtained *Q*_∞_ and *K*_is_ data of PAA/SC, the initial swelling rate can be obtained in the following order: PAA/SC (10 wt%) > PAA/SC (14 wt%) > PAA/SC (18 wt%) > PAA/SC (6 wt%) > PAA/SC (2 wt%) > PAA/SC (0 wt%) in distilled water and PAA/SC (10 wt%) > PAA/SC (18 wt%) > PAA/SC (6 wt%) > PAA/SC (14 wt%) > PAA/SC (2 wt%) > PAA/SC (0 wt%) in 0.9 wt% NaCl solution, respectively. The results indicated that the modest introduction of SC into PAA system could improve the swelling rate of superabsorbent composites. The reason was the coarse surface of the superabsorbent composites will accelerate the diffusion of water molecular into the matrix of PAA/SC. In addition, the extended three-dimensional network and the weaker chain entanglement of PAA/SC also sped up the swelling process.

#### 3.3.7. Water-Retention Capacity at Room Temperature

**Figure 7:**
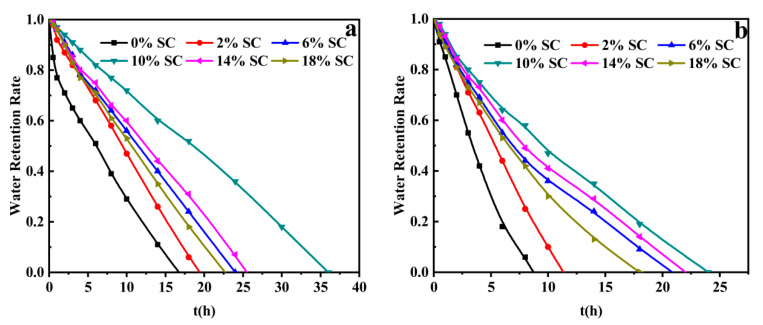
(**a**) Water-retention behaviors of PAA/SC prepared by different content of SC with the swelling equilibrium in distilled water at room temperature (7.2 g AA, 0.4 mol% MBA, 1.60 mol% APS and neutralization degree 60% of AA were selected and prepared superabsorbent composites with SC content 0 wt%, 2 wt%, 6 wt%, 10 wt%, 14 wt% and 18 wt%, respectively); (**b**) Water-retention behaviors of PAA/SC prepared by different content of SC with the swelling equilibrium in 0.9 wt% NaCl solution at room temperature (7.2 g AA, 0.4 mol% MBA, 1.60 mol% APS and neutralization degree 60% of AA were selected and prepared superabsorbent composites with SC content 0 wt%, 2 wt%, 6 wt%, 10 wt%, 14 wt% and 18 wt%, respectively).

Figure 7 presented the water-retention capacity of PAA/SC with the swelling equilibrium at room temperature. About 50 mg of the dried superabsorbent composites samples were immersed in 400 mL of distilled water and 400 mL 0.9 wt% NaCl solutions to reach the swelling equilibrium for 4 h, respectively. At room temperature, the swollen samples were placed in glass dishes and exposed to air. The PAA, which was swollen in distilled water and 0.9 wt% NaCl solutions needed 17 h or 8.5 h to lose all the absorbed water, respectively. While the water-retention capacity of superabsorbent composites of PAA/SC (10 wt%) had an understanding extension, needing 36 h and 23 h, respectively. This result indicated that the introduction of SC into PAA system could improve the water-retention capacity of superabsorbent composites. This feature of the water-retention capacity may be exploited so that the addition of PAA/SC samples in the soil decrease water evaporation. The PAA/SC will endow the soil with excellent water-retention capacity and is a potential water-retaining agent used in the agriculture.

#### 3.3.8. Reswelling Capability

**Figure 8:**
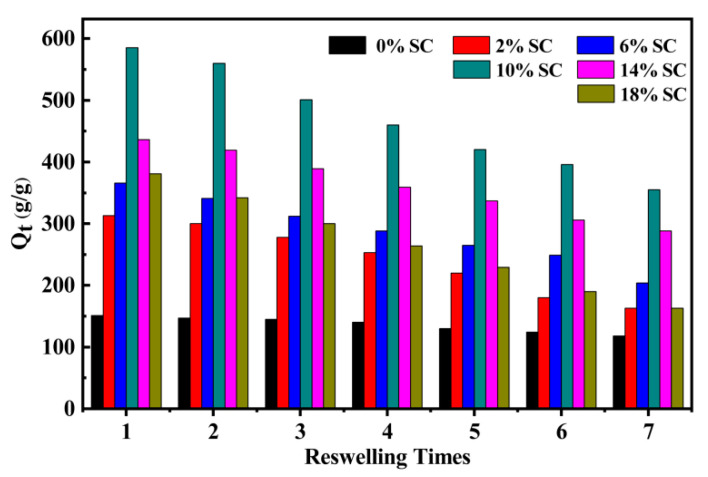
Water absorbency of PAA/SC prepared by different content of SC as a function of reswelling times (7.2 g AA, 0.4 mol% MBA, 1.60 mol% APS and neutralization degree 60% of AA were selected and prepared superabsorbent composites with SC content 0 wt%, 2 wt%, 6 wt%, 10 wt%, 14 wt% and 18 wt%, respectively).

The dry superabsorbent composites still displayed a better water-absorbing capability than the composites without SC, while the fully swollen superabsorbent was completely dehydrated at 90 °C in a vacuum oven. Figure 8 showed the reswelling capability for PAA/SC superabsorbent composites as a function of reswelling times in distilled water. It can be seen that the PAA/SC (14 wt%), PAA/SC (10 wt%) and PAA/SC (6 wt%) showed good reswelling capability and still retained approximately 66.06%, 61.68% and 55.74% of their initial water absorbency after re-swelling seven times [48]. These results suggested that the superabsorbent composites of PAA/SC were reusable and recyclable water-absorbing materials, and can be especially useful in agricultural applications. In addition, it also showed that SC could obviously prolong utilization periods of PAA/SC.

## 4. Conclusions

As part of the efforts to reduce excessive environmental pollution and explore potential application value for oil shale SC, a series of PAA/SC superabsorbent composites were synthesized in one step by polymerization of AA using APS as an initiator and MBA as a crosslinker, as well as SC micropowder as inorganic fillers and graft copolymers. FTIR and SEM analysis indicated that the superabsorbent composites have been successfully prepared by free-radical polymerization and the shatter value of polymeric structure increased with the increase of SC content. Meanwhile, the factors of effect on water absorbency, such as content of initiator, crosslinker, SC, and the neutralization degree were investigated. Under optimal synthesis conditions, it was shown that the superabsorbent composites displayed the best water absorbency of 584 g/g and 75 g/g in distilled water and in 0.9 wt% NaCl solution, respectively, as the 10 wt% of SC was introduced into the PAA/SC. Besides, the superabsorbent composites had the high water absorbency in the pH range of 4–11, which was advantageous for their potential application in agriculture. The swelling kinetics of PAA/SC obey Scott’s kinetic model in distilled water and in 0.9 wt% NaCl solution, and the initial swelling rate constant reached the maximum value with the SC content 10 wt%. After reswelling seven times, the superabsorbent composites of PAA/SC (14 wt%) showed good reswelling capability and still retained approximately 66.06%. The experiment result reported in this study not only realized the waste utilization and effectively reduced the product cost, but also integrated the excellent water absorbing capability and reswelling properties; the superabsorbent composites can be used as potential water-retaining agent in agricultural applications.

## Figures and Tables

**Table 1:** Swelling kinetic parameters of PAA/SC prepared by different content of SC in distilled water (7.2 g AA, 0.4 mol% MBA, 1.60 mol% APS and neutralization degree 60 % of AA were selected and prepared superabsorbent composites with SC content 0 wt%, 2 wt%, 6 wt%, 10 wt%, 14 wt% and 18 wt%, respectively).

Samples	*Q*_eq_ (g/g)	*Q*_∞_ (g/g)	*K*_is_ (g/g·s)	*K*_s_ (× 10^−5^, g/g·s)
PAA/SC (0 wt%)	151	158	1.6009	6.4128
PAA/SC (2 wt%)	313	320	3.9246	3.8326
PAA/SC (6 wt%)	366	378	4.3605	3.0518
PAA/SC (10 wt%)	584	592	6.6798	1.9060
PAA/SC (14 wt%)	436	441	5.1533	2.6498
PAA/SC (18 wt%)	381	398	4.7455	2.9958

**Table 2:** Swelling kinetic parameters of PAA/SC prepared by different content of SC in 0.9 wt% NaCl solution (7.2 g AA, 0.4 mol% MBA, 1.60 mol% APS and neutralization degree 60% of AA were selected and prepared superabsorbent composites with SC content 0 wt%, 2 wt%, 6 wt%, 10 wt%, 14 wt% and 18 wt%, respectively).

Samples	*Q*_eq_ (g/g)	*Q*_∞_ (g/g)	*K*_is_ (g/g·s)	*K*_s_ (× 10^−4^, g/g·s)
PAA/SC (0 wt%)	46	48	0.5256	2.2812
PAA/SC (2 wt%)	60	63	0.6646	1.6745
PAA/SC (6 wt%)	67	68	0.7205	1.5582
PAA/SC (10 wt%)	75	78	0.7798	1.2817
PAA/SC (14 wt%)	69	70	0.7033	1.4353
PAA/SC (18 wt%)	63	65	0.7455	1.7645

## Supplementary Materials

The following are available online at https://www.mdpi.com/2073-4360/12/10/2347/s1, Figure S1: XRD characterization of SC, Figure S2: TG characterization of SC and PAA/SC, Figure S3: Elemental mapping images for C, O, Si, Al and Fe within the as-prepared PAA/SC, Table S1: The sample yield for each condition.
