# Supplementary material for: Fabrication of Eco-Friendly Superabsorbent Composites Based on Waste Semicoke
Source: Polymers (Basel). 2020 Oct 14;12(10):2347. doi: 10.3390/polym12102347 (PMC7602290; doi:10.3390/polym12102347)
Supplement: Supplementary file 1 [file polymers-12-02347-s001.pdf]

Fabrication of eco-friendly superabsorbent composites based on waste semicoke

Yongsheng Wang[1, 2], Yongfeng Zhu[1], Yan Liu[1] and Aiqin Wang[1,*]

[1] Key Laboratory of Clay Mineral Applied Research of Gansu Province, Center of Eco-material and Green Chemistry, Lanzhou Institute of Chemical Physics, Chinese Academy of Sciences, Lanzhou 730000, P.R. China; wysh0304@126.com(Y.W.);zhuyf851013@163.com (Y.Z.);1764369602@qq.com (Y.L.)

[2] Center of Materials Science and Optoelectronics Engineering, University of Chinese Academy of Sciences, Beijing 100049, P.R. China;wysh0304@126.com (Y.W.)

* Correspondence: aqwang@licp.cas.cn; Tel.: +86-931 4968118

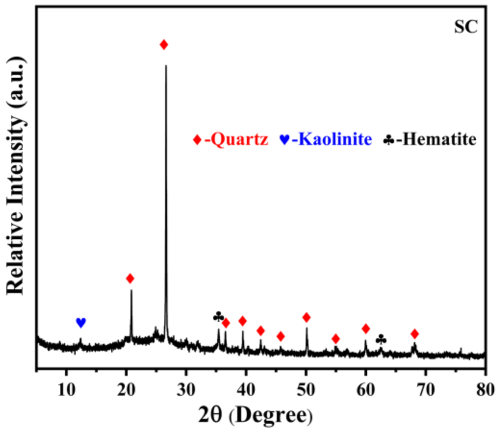

Figure S1 XRD characterization of SC

As shown in **Figure S1**, The XRD characterization revealed that the crystal phase ingredients of in the SC mainly include quartz (characteristic reflections at 2θ = 20.82°, 26.60°, 36.54°, 39.46°, 42.45°, 54.87°, 59.96° and 68.36°), kaolinite (characteristic reflections at 2θ = 12.36° (0 0 1 crystal plane) and 2θ = 19.84° (0 2 0 crystal plane)) and hematite (characteristic reflection at 2θ = 35.61°, 62.32 °).

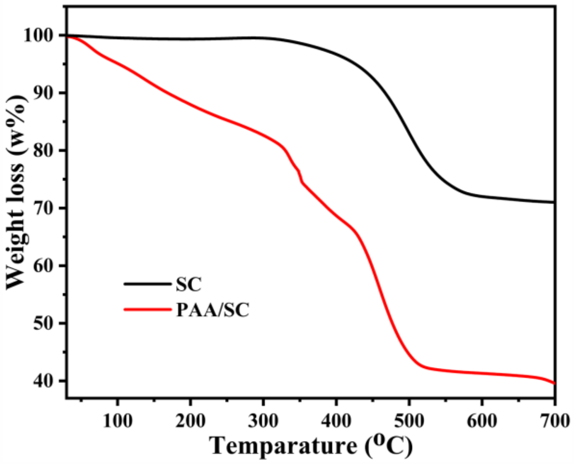

Figure S2 TG characterization of SC and PAA/SC

As shown in **Figure S2**, SC had two significant weight loss stages, including 30 to 100 ℃ and 350 to 600 ℃, with weight loss of 1.8% and 27.3%, respectively. The first stage of weight loss was mainly attributed to the loss of absorbed water and bound water. The second stage of weight loss was due to the degradation of carbonic matter in SC. PAA/SC had three distinct weight loss stages, the weight loss in the first and third stages were almost identical to that of SC. The second stage of weight loss from 150 to 350 ℃ may be attributed to the decomposition of different structure of the graft copolymer.

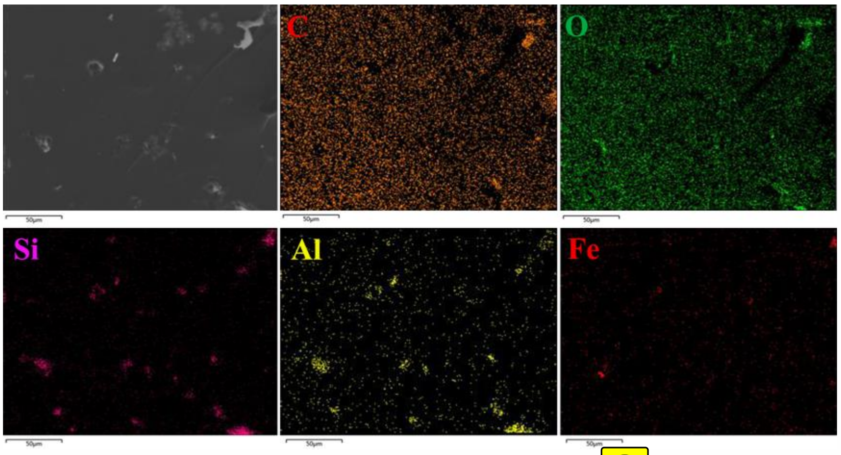

Figure S3 Elemental mapping images for C, O, Na, S, Si, Al and Fe within the as-prepared PAA/SC

Table S1 The sample yield for each condition

Selected conditional		The yields of the products
Effects of APS content on the water absorbency (7.2 g AA, 800 mg SC, 0.4 mol% MBA and neutralization degree 70 % of AA were selected)	APS (1%)	95.2%
	APS (1.2%)	93.5%
	APS (1.4%)	94.8%
	APS (1.6%)	92.5%
	APS (1.8%)	94.2%
	APS (2%)	96.6%
Effects of MBA content on the water absorbency (7.2 g AA, 800 mg SC, 1.60 mol% APS and neutralization degree 70 % of AA were selected	MBA (0.2%)	92.1%
	MBA (0.3%)	97.3%
	MBA (0.4%)	95.5%
	MBA (0.5%)	96.4%
	MBA (0.6%)	93.7%
	MBA (0.7%)	93.4%
Effects of the neutralization degree on the water absorbency (7.2 g AA, 800 mg SC, 0.4 mol% MBA and 1.6 mol% MBA were selected	50% of AA	96.6%
	55% of AA	95.2%
	60% of AA	94.4%
	65% of AA	96.1%
	70% of AA	97.3%
Effects of SC content on the water absorbency (7.2 g AA, 0.4 mol% MBA, 1.60 mol% APS and neutralization degree 60 % of AA were selected	SC (0%)	95.8%
	SC (2%)	96.4%
	SC (6%)	96.8%
	SC (10%)	96.1%
	SC (14%)	95.8%
	SC (18%)	97.3%
